# Medical Opinion and Movement

**Published:** 1911-05-06

**Authors:** 


### Medical Opinion and Movement.

### Dislocation of the Shoulder.

In the *Revue de Chirurgie* Drs. Nubert and Dugas, of Marseilles, discuss the ultimate prognosis of dislocation of the shoulder-joint based upon fifteen old cases of dislocation, thirteen subcoracoid, and two subglenoid, that had been readily reduced shortly after the accident. All complicated cases were excluded, in order to ascertain the final result of simple shoulder dislocations. Such cases are usually followed by functional impairment and painful symptoms for many months. This was the case with nearly all the patients. Temporary incapacity may become permanent in spite of all efforts to prevent it. It is, therefore, unwise to state what the results of a shoulder dislocation will be until several months have passed. Only exceptionally an impairment of function depends upon traumatic paralysis of parts supplied by branches of the brachial plexus. Periarthritis of the shoulder-joint is the most common cause of the sequelæ of dislocation. Peripheral neuritis is a less common cause. In some cases incapacity seems to depend upon the development of subacromial bursitis. Curiously, the authors do not refer to the liability of recurrence of the dislocation.

### Pulmonary Tuberculosis.

Dr. Roubel has carried out some interesting experiments to test the validity of the notion that functional rest of a tuberculous lung is favourable to a cure. He did not resort to the production of an artificial pneumothorax, as the functional rest for the lung obtained in this way is accompanied by accessory factors such as mechanical compression, the effect of which it is impossible to estimate separately. The method adopted was as follows: An incision was made over one of the ribs of the rabbit in the submaxillary line, dividing the skin and subcutaneous tissue. A metallic filament was then carried by means of a needle through one intercostal space, piercing the pleura, and out again through another intercostal space, so as to embrace two or three ribs. The ends of the filament were then pulled together and tied, so as to draw the enclosed ribs closely together. The skin incision was sutured and a collodion dressing applied. Six to nine days later an emulsion of tubercle bacilli was injected into the auricular vein. Microscopic examination showed that the bacilli accumulated in greater numbers and more closely in the lung on the immobilised side than in the opposite lung, the anatomical lesions were more serious and more extensive, infiltration more intense, hepatisation greater, and a larger formation of tubercles. Such was the early condition; but in preparations from more advanced cases, especially after six weeks, the diffuse cellular infiltration was less marked on the immobilised side and the separation of the tubercles greater. Further, the process of fibrous transformation of the tubercles appeared much more energetic on the immobilised side. Dr. Roubel concludes, therefore, that functional rest favours a re-absorption of the inflammatory tuberculous infiltrations and the fibrous transformation of the tubercles. On the other hand, bacilli entering by means of the blood-vessels tend to accumulate in much greater quantities in the tissues of a lung more or less at rest than in those of a lung functionating in a normal manner.

### Fractures and Osmic Acid.

Italian surgeons report some very satisfactory results in cases of ununited fractures and pseudo-arthroses by injections of osmic acid. Dr. Novaro first hit upon the idea from the result he obtained in a case of spontaneous fracture of the femur, supposed due to sarcoma. The osmic acid injections were made according to the method of Winiwarter as a specific treatment for the sarcoma. Complete consolidation took place, but as the treatment failed in subsequent cases of sarcoma, Dr. Novaro concluded that the first case of spontaneous fracture was due to a syphilitic gumma. Some years later he applied the same treatment to a case of fractured femur in an officer, which failed to unite. The probability of syphilis was admitted, but specific treatment had no effect. Osmic acid injections speedily effected a cure. Dr. Zanardi reports two cases of ununited fracture successfully treated in the same way, and Dr. Onorato more recently gives an account of five cases, two cases of pseudo-arthrosis and three cases of ununited fracture, in all of which osmic acid injections resulted in rapid consolidation of the bones. In all these cases there was a history of syphilis, but specific treatment with mercury and iodides failed to produce any curative effect. The osmic acid injections are made at the seat of the injury, and about 0.5 c.c. of a 1 per cent. aqueous solution of osmic acid is injected, the injection being repeated in seven to ten days. Two or three injections are usually sufficient.

Dr. Onorato has carried out some experiments on animals to determine the mechanism of the action exercised by the osmic acid. He finds that the injection of one drop of a 1 per cent. solution of osmic acid at the periosteum of the femur causes the formation of an exostosis at the point where the periosteum has been irritated. If, on the other hand, a considerably larger quantity is injected and still more if a stronger solution is used, necrosis of the periosteum and even of the subjacent osseous lamellæ takes place. Experimental fractures in which a small quantity of the 1 per cent. osmic acid solution was injected, healed more rapidly than those in which no such injections were made. In regard to the apparent connection between syphilis and delay in consolidation of the bone, the author was unable to obtain any experimental confirmation. Rabbits were inoculated with syphilitic virus, but there was no delay in the healing of fractures subsequently produced; but these negative results do not prove that syphilitic infection is not an obstacle to the normal healing of fractures, and clinical observation certainly points to the opposite conclusion. The experimental work shows that the injections should be limited to small doses of a weak solution of the acid. They should be made so that the solution can spread along the seat of the fracture. Large doses may produce necrosis and also a severe general reaction. In Dr. Zanardi's first case he injected 5 c.c., and this was followed by a violent local and general reaction, with extreme pain and fever.

### Salicylates by Subcutaneous Injection.

Dissatisfied with some of the results of salicylate administration by the mouth in cases of acute rheumatism, Dr. Seibert four years ago began to experiment with subcutaneous methods of introducing them into the system. He now adheres to the following rules, which have been evolved as the result of experience. In acute rheumatic infection of joints, heart, pericardium, pleura, and central nervous system, 10 c.c. of a 20 per cent. sterilised solution of fresh salicylate of soda are used per 100 lb. of body weight. This is repeated every twelve hours; and in very bad cases, half as much again has been given. A spot outside the median line of the thigh is disinfected with tincture of iodine; through this, thirty drops of sterilised cocaine solution (containing $\frac{1}{8}$ grain of the alkaloid) is injected under the skin. In fifteen minutes the salicylate solution is injected, care being taken that none of it is pressed into the skin, but that it is placed well into the subcutaneous tissues. In chronic cases an oily solution of salicylic acid is used instead of the watery solution of salicylate of soda. It is said that marked improvement is often visible within three hours of the first injection. The addition of 5 to 20 per cent. of camphor has also been found beneficial in stimulating the heart, and especially in carditic cases. One of the most valuable properties claimed for this subcutaneous treatment is the entire absence of toxic symptoms.

### A Censor of Asepsis.

In the *Medical Record* Dr. Dawbarn has been publishing recently a series of short talks and random suggestions on many topics connected with surgery. One of his interesting paragraphs deals with an institution in his hospital clinic known as the Censor of Asepsis. This officer is an experienced interne, who stands on a stool in the operating theatre and devotes his entire attention to the detection of breaches of aseptic technique. Surgeon, assistant, nurses, students—all are closely watched, and the very slightest infraction of asepsis is immediately reported, even though the offender be the operator himself. Dr. Dawbarn is at pains to defend the last extreme of rigour in the operating theatre of a teaching surgeon: thus the avoidance of the slightest crevice or cross-hatching on a knife or other tool is a duty on which he insists, not so much because it is really essential, but because of the good example of thoroughness. In his succeeding short talk, the author deals briefly with the use of egg-membrane for skin-grafting. Place upon the wound, he says, having first made it aseptic and free from granulations as in the Thiersch method, that surface of the egg-membrane which normally lies deepest in the egg. The other side, that which lies in contact with the shell, alone has the epithelial cells which are necessary for cuticle formation. A pullet's egg is not to be used, but that of a hen: the reason is that the stouter shell of a hen's egg keeps the membrane aseptic much more effectively than the thinner and more porous shell of a pullet's egg.

### Nature and Treatment of Chorea.

In a very able paper contributed to the *Bristol Medico-Chirurgical Journal*, Dr. Carey Coombs summarises his clinical observations and post-mortem researches into chorea. He formulates his series of propositions thus: Sydenham's chorea is a manifestation of rheumatic infection. It is an organic disease of the brain, attacking all parts of the cortex equally and impartially, but affecting the basal grey matter less severely. Consequently, the symptoms are not only motor, but also psychical, and sometimes sensory. The motor symptoms are partly due to loss of inhibition, partly to cortical irritation. In chorea the fallacy of the distinction between "functional" and organic nervous disease is exemplified. Chorea can be fatal without the occurrence of meningeal inflammation; and this fact, with the phenomena of latent chorea, suggests that chorea may be due to direct intoxication rather than to gross infection of the cranial contents. The tendency of chorea is towards restoration of health to the brain. Treatment consists of (*a*) removal of the cause (active rheumatic infection) by rest in bed with administration of salicylates during the active stages; (*b*) prolonged mental and bodily rest during convalescence; (*c*) improvement of general health by fresh air, full diet, and tonics; (*d*) quieting of excessive movement by sedative drugs or packs, and cure of paresis by massage. The full-dose arsenical treatment is useless. Chloretone is only useful in certain cases, and should not be given as a routine measure.

### A Method of Outlining the Viscera.

Dr. Creighton Wellmann describes in the *Interstate Medical Journal* a procedure whereby the size and position of various viscera such as the heart, spleen, and liver, and also tumours, can be accurately determined. For example, if it is decided to map out the position of the liver, a stethoscope is placed over the centre of the area of liver dulness. A large tuning-fork is next obtained and made to vibrate by striking it against some hard object such as the side of the bed. Whilst vibrating the handle of the fork is placed on the surface of the body at a point beyond the border of the liver area, and then moved slowly towards the stethoscope until the singing-note of the fork is heard. The author claims that if this manœuvre be repeated in all directions the organ can be outlined with almost mathematical exactness. He warns his readers however that bone will transmit the sound, so that in working down the back and chest the handle of the fork should not be placed on the ribs.

### Ether Compresses.

Dr. Pietro Franzoni, of Brescia, relates in *La Clinique* how he came to use compresses soaked in ether in cases of contusions and wounds rendered extremely painful by circulatory disturbances. The fact that œdema and tumefaction were sometimes added further to compromise tissue vitality seemed to him an additional reason for making use of this treatment. To his surprise and satisfaction, as well as that of his patients, he found that compresses of sulphuric ether always led to a prompt resorption of extravasated blood, a rapid diminution of the congestion with relief of pain, and marked improvement of the injured parts. The general condition of the patient is influenced satisfactorily also. Such compresses cause the disappearance of tumefaction and pain in cases of fracture and allow of easy reduction. In cases of peritonitis, apoplexy, phlegmon, uterine hæmorrhages, erysipelas, periostitis, synovial effusions, orchitis, iritis, tonsillitis, etc., the decongestionising power of the drug is marked if applied early in the disease. The ether may be applied pure or after mixing it with goulard or hydrogen peroxide. It is sufficient to soak compresses in the fluid and to apply them directly to the affected parts. As soon as dry they should be soaked again in the fluid and reapplied as long as considered necessary. The indication for their removal rests for the most part with the patient, who will declare that his pain is relieved. When dealing with open wounds, these should be covered with some waterproof material before applying the compresses. There is no harm done, however, if the ether should happen to reach the tissues. This form of treatment is superior to that by the ice-bag, as the latter weighs heavy. Ether is at once antiseptic, anæsthetic, and hæmostatic; the one disadvantage to its use lies in the fact that it is extremely inflammable, and consequently great care must be exercised to keep naked lights out of its reach.

### Radium Water Therapy.

It is generally agreed that "Indifferent Thermal" waters produce their effects as the result of radio-activity. This last is, however, so small as to be difficult of estimation until Macé of Vienna invented a unit—one-thousandth part of an ordinary electro-static unit. The amount of radio-activity present in various natural waters varies from 2 to 150 of these units. In 1905 Gottlieb found marked medical results amongst miners who worked at Jacomsthal, the great Austrian radium mine, and more so when they drank the mine water. Dautwitz added pitchblende to existing indifferent thermal waters with enhanced results. Dr. William Armstrong, of Buxton, who gives these facts in an article in the *British Medical Journal* under the above heading, showed some eight years ago, along with Dr. Harburn, that the action of Buxton waters was almost entirely due to contained gases, and in 1910 began to administer much stronger radioactive water. He now published results obtained in a series of 400 cases. The general physical results obtained were as follows: (1) Greatly increased diuresis; (2) Increased excretion of carbonic-acid gas; (3) Marked lowering of blood-pressure, especially in arterio-sclerosis; (4) Improved digestion; (5) Disappearance of gouty deposits; (6) Increased activity; (7) Regulation of bowel activity without purgation; (8) Great improvement in appearance. In special cases his results were as follows: Diabetes, decrease of sugar, general improvement. Glycosuria, rapid decrease and complete disappearance of sugar. Albuminuria, decrease of albumin and blood pressure; improvement in general health. Arteriosclerosis, decrease in blood pressure from 20 to 80 mm. Gout, urine increased with uric acid excretion; parts affected relieved. Rheumatism, pain and stiffness relieved. Neuritis, pain relieved; great improvement in nerve health. Arthritis, improvement in nearly all forms, especially when recent non-acute. Neurasthenia, improvement most satisfactory of all in this group. Impaired sexual vitality, good results in twelve cases. Sympathetic nerve affections, especially vaso-motor troubles, made very satisfactory progress.

### Salvarsan.

Among the host of papers dealing favourably and adversely with this much-exploited remedy, a very interesting one of the former class is that contributed to the *Journal of the Royal Army Medical Corps* by Major Gibbard, Captain Harrison, and Lieutenant Cane. It is important to notice that these authorities have entirely abandoned the subcutaneous method of administration, on account of the not very infrequent rise of sloughing afterwards, sometimes at considerable intervals of time. Thus, in one case where the tissues at the site of inoculation sloughed four and a half months after injection, arsenic was found in considerable quantity by chemical analysis in the sloughs. The intravenous method has the sanction of Professor Ehrlich, and in the great majority of cases no after-results of an unpleasant sort are evident; in a very few cases some nausea and vomiting occurred. When thus used, the authors conclude that salvarsan has a marked and rapid effect on the various manifestations of syphilis. This effect is distinctly more rapid than that following the administration of mercury, and is often very pronounced when the latter drug has failed to produce any impression on the disease. Various observations indicate, they believe, that the clinical effects are due to a specific action of the drug on the parasites, and therefore that treatment by salvarsan is not merely symptomatic. Whether this action consists in destruction of the parasites or is simply a temporary suppression of their activity they are not prepared to say. In view of these conclusions, they feel justified in pronouncing that salvarsan is a valuable remedy in the treatment of syphilis, and that adverse criticism on the score of relapses will be unjustifiable until the best method of administering it has been discovered.

